# Treatment of canine otitis externa: a randomized, double-blinded, clinical field study comparing the hydrocortisone aceponate monotherapy to a multi-agent reference product

**DOI:** 10.3389/fvets.2026.1861746

**Published:** 2026-07-28

**Authors:** Tomoko Kuwahara, Laure Poincelot, Pierre Jasmin, Alice Bidaud, Gen Kinoshita

**Affiliations:** 1 Nippon Virbac Co. Ltd, Tokyo, Japan; 2 Virbac SA, Carros, France

**Keywords:** atopic dermatitis, cortotic, dog, hydrocortisone aceponate, osurnia, otitis

## Abstract

Erythemato-ceruminous otitis externa (ECOE) is a canine inflammatory condition very frequently involving secondary microbial infections. This multicentric, masked, randomized, clinical field trial in Japan evaluated the efficacy and safety of a hydrocortisone aceponate (HCA) spray solution (Cortotic™, Virbac, France) compared to a terbinafine/florfenicol/betamethasone acetate ear gel (Osurnia™, Bussan Animal Health CO Ltd, Japan) in 200 dogs with ECOE (OTIS3 ≥ 5). Dogs received either Cortotic™ daily for 7 or 14 days or Osurnia™ on Days 0 and 7. OTIS3 clinical evaluation and cytological examinations were conducted, as well as the subjective veterinarian product efficacy evaluation. Results showed no significant differences between groups in OTIS3 score reduction, cytological reduction, or treatment success rates at any time point. On Day 28, Cortotic™ showed a 72.1% OTIS3 reduction, comparable to Osurnia™ 76.1%. Treatment success rates were also similar, and relapse rate was low in both groups. On Day 28, the veterinarians subjectively assessed the efficacy as good or excellent in 83% of cases for Cortotic™ and 87% for Osurnia™. Both treatments were well-tolerated. Cortotic™ demonstrated non-inferiority to Osurnia™ regarding clinical and cytological efficacy. The use of Cortotic™, a corticosteroid-monotherapy, can offer an effective strategy for managing ECOE while avoiding unnecessary antibiotic exposure. This study supports Cortotic™ as a first-line therapeutic option for otitis externa in dogs.

## Introduction

Canine otitis externa is a common, often recurrent, inflammatory disorder of the external ear canal. Achieving a long-term clinical remission requires addressing the multifactorial etiology of the disease using the primary-secondary-predisposing-perpetuating framework (1). Inflammatory disorders, particularly canine atopic dermatitis, represent the most frequent primary causes of otitis externa, though foreign bodies, parasites, endocrinopathies, tumors, and other rare affections can also initiate the condition. Predisposing factors may increase the likelihood of otitis externa development or progression by altering the environment of the ear canal. These include anatomical and conformational traits such as stenotic ear canals, pendulous pinnae, increased density of ceruminous glands, as well as lifestyle influences including excessive moisture from swimming or iatrogenic damage from improper cleaning techniques. Once primary inflammation is established, secondary factors complicate the clinical presentation. Notably, recurrent microbial overgrowths are secondary phenomena, typically starting with a dysbiosis of the local microbiome rather than the acquisition of a novel pathogen. This dysbiosis frequently manifests as an overgrowth of *Malassezia* yeasts, and/or cocci bacteria in erythemato-ceruminous otitis externa (ECOE). If these underlying primary inflammation and its secondary issues are not comprehensively managed, perpetuating factors can be induced. These consist of chronic, acquired pathological changes in the ear canal, initiating as nodular epidermal and ceruminous gland hyperplasia which may progress to severe ear canal stenosis, occlusion, fibrosis, and dystrophic mineralization (1–4).

Historically, treatment protocols have quasi-systematically involved products combining anti-inflammatory, antibacterial and antifungal agents with various frequency and duration of administration. More recently, single-use formulations allowing for single administration have been introduced, offering owners a simpler treatment option. This ear medication maintains the therapeutic concentration of active ingredients in the ear canal without relying on daily administration by the pet owner (5). An innovative therapeutic approach involves the exclusive use of an anti-inflammatory agent, targeting the primary drivers of ECOE (6, 7). Hydrocortisone aceponate (HCA), a potent and topically active corticosteroid, is characterized by its rapid metabolism into inactive metabolites, thereby minimizing systemic exposure. Its application in veterinary medicine is well-established for the management of canine atopic dermatitis (6, 8, 9). A prior investigation compared the administration of hydrocortisone aceponate alone with a conventional product containing anti-inflammatory, antimicrobial, and antifungal components (Surolan™, Elanco, Germany). This study revealed high efficacy of the hydrocortisone aceponate-only product (Cortotic™, Virbac, France) with no significant difference in clinical treatment between the two treatment groups. These findings offer encouraging perspectives for refining the management of ECOE, particularly within the context of responsible antimicrobial stewardship (7). However, the clinical significance of these observations necessitates further investigation within a broader clinical context. The present study was designed to evaluate the efficacy and safety of a hydrocortisone aceponate spray solution (Cortotic™, Virbac, France) through a comparative study with another combination product, a terbinafine/florfenicol/betamethasone acetate ear gel (Osurnia™, Bussan Animal Health CO Ltd, Japan) in client-owned dogs presenting with otitis externa.

## Materials, animals and methods

This was a multicentric, double-blinded, randomized, parallel-group field trial. The hypothesis to be tested in the present study is that Cortotic™ administered once a day for a minimum of 7 days and up to 14 days, is non-inferior to Osurnia™ administered twice with a 1-week interval on Days 0 and 7. The non-inferiority is based on the percentage of change from baseline in OTIS-3 at the end of the study (D28). The study was masked by function. Cortotic™ and Osurnia™ have different appearances. Therefore, to preserve masking, a “Dispenser” was identified at each veterinary practice for the allocation and dispensing of study treatments. The veterinarian responsible for the efficacy and safety evaluations was masked during the whole study. He did not have access to information that might give him any details of the treatment groups. The study was conducted in compliance with Good Clinical Practice (GCP) guidelines.

### Ethics

This clinical trial was conducted with the highest priority given to the human rights of the owners and the welfare of animals. Participation in the clinical trial was based on the voluntary intention of the owners informed orally and in writing about the content of the clinical trial and the rights. The Ethical Review Committee of SHOKU-KAN-KEN Inc. gave a positive opinion with the approved number AW21Apr003H.

### Animals

The study was carried out in 10 veterinary clinics distributed in Japan. Client-owned dogs, of at least 4 months old, exhibiting clinical signs of non-parasitic otitis externa with an Otitis Index Score (OTIS3) of 5 or greater were enrolled in the study (10). On Day 0, a cytological examination of the ear samples was performed by the veterinarian to confirm the presence of bacteria and/or yeast and the absence of parasites.

The exclusion criteria consisted of a ruptured tympanic membrane, purulent exudate, otic ectoparasites, or a foreign body identified during the initial otoscopic examination.

### Treatment

Dogs were randomly assigned to one of two treatment groups. At each study site, treatment was allocated according to a unique block randomization sequence generated specifically for each veterinary clinic.

Prior to the initial treatment administration on Day 0, the affected ear canal in all dogs was cleaned using a commercially available ear cleaner (e.g., EpiOtic™, Virbac, France) (11). No further ear cleansing was permitted during the study period.

One group of dogs received Cortotic™ (Virbac, France), a hydrocortisone aceponate spray solution administered as two pump sprays into the affected ear canal once daily for 7 or 14 days. If the OTIS-3 clinical score remained ≥4 on Day 7, treatment was extended for an additional 7 days.

The other group received Osurnia™ (Bussan Animal Health CO Ltd, Japan), a gel containing betamethasone acetate, florfenicol, and terbinafine, administered as 1 ml into the affected ear canal on Day 0 and Day 7.

All dogs were examined on D14 and, independently of the scoring obtained on the OTIS-3, treatment was ended on D14.

Both products were administered in accordance with the respective manufacturer's recommendations.

### Safety evaluation

Safety was assessed by monitoring for any local or systemic adverse reactions throughout the study period. All dogs receiving at least one dose of a study product were included in the safety evaluation. At each scheduled visit, a general physical examination was conducted by the attending veterinarian. Furthermore, owners were instructed to observe their dogs daily for any health-related changes and to report any observed abnormalities without delay.

### Monitoring and samples

Clinical assessments were conducted on Days 0, 7, 14, 28, and 42.

The clinical severity of otitis externa was evaluated by a board-certified veterinarian using the OTIS3 scoring system. This system is an adaptation of the canine otitis externa clinical scoring index described by Nuttall and Bensignor (10). The OTIS3 index evaluates four distinct clinical parameters: erythema, edema, exudate (discharge), and ulceration (erosion). Each criterion is graded on a scale of 0–3, with the final OTIS3 score representing the cumulative sum of these individual values (0–12).

The veterinarian provided a subjective global assessment of treatment response, ranging from excellent to poor, based on the observed improvement in clinical signs compared to the baseline examination.

Ear swabs were collected by the veterinarians at each study day. Samples were submitted to a laboratory for semi-quantitative cytological evaluation. Cytological assessment was performed using the validated Budach and Mueller scale (12). This 5-point grading system was employed to semi-quantitatively evaluate the presence and density of 3 categories: bacteria, yeast and inflammatory cells. Each category is assigned a grade on a scale from 0 to 4+ based on the quantity of the elements and how easily they can be found on the cytological preparations. A score of 0 indicates that no bacteria, yeast, or inflammatory cells are present. A score of 1+ is given when there are occasional elements present, but the examiner must scan the slide carefully to detect them. A score of 2+ indicates that elements are present in low numbers but can be found rapidly and without difficulty. A score of 3+ is assigned when there are larger numbers present that are rapidly detectable. Finally, a score of 4+ represents massive amounts of bacteria, yeast, or inflammatory cells that are rapidly and easily detectable. The total cytological score is calculated by summing the individual grades assigned to each evaluated categories.

Treatment success was defined as achieving an OTIS3 score ≤ 3 and was assessed at D7, D14, D28 and D42. The relapse rate was evaluated by calculating the percentage of dogs with an OTIS3 score ≥4 at Day 42 among those having a treatment success at Day 28.

### Statistical analysis

Statistical analyses were performed using SAS software (version 9.4 or higher). The primary non-inferiority assessment was based on the percentage change from baseline in the OTIS-3 clinical scoring system at Day 28. The sample size was based on a previous study (5) where the mean percentage change from baseline in OTIS-3 at Day 28 for Osurnia was estimated at 63% (*SD* 30%). Setting the non-inferiority margin at 15% (representing 2 points on a 0–12 scale), a minimum of 85 evaluable dogs per group was required to achieve 90% power at a two-sided 5% significance level. To account for potential attrition, 200 dogs were randomized (*n* = 100 per group). Non-inferiority was established if the lower bound of the two-sided 95% confidence interval (CI) for the treatment difference was greater than −15%. Upon confirmation of non-inferiority, a subsequent test for superiority was performed at a two-sided 5% significance level.

## Results

### Population

A total of two hundred dogs (200) were enrolled and randomly assigned to two treatment groups (*n* = 100 per group). All 200 dogs were included in the safety and efficacy analyses through Day 28. In accordance with the protocol, the requirement for continued Cortotic treatment was assessed at Day 7 based on clinical success (OTIS 3 <3). Consequently, in the Cortotic group, only 37 dogs out of 100 required an additional 7 days of treatment; completing a total treatment course of 14 days. In contrast, all dogs in the Osurnia group received a full 14-day treatment duration via the mandated two-dose regimen on Days 0 and 7. On Day 28, 48 dogs (Cortotic: *n* = 26; Osurnia: *n* = 22) were withdrawn from the study due to treatment failure. Consequently, 152 dogs (Cortotic: *n* = 74; Osurnia: *n* = 78) were evaluated at the Day 42 assessment. No protocol deviations were reported during the study period.

Baseline demographic characteristics were comparable between groups regarding breed distribution, age, sex, and body weight. The overall mean age of the study population was 4.6 ± 2.4 years (range: 1–12 years). The mean body weight was 10.0 ± 7.3 kg (range: 1.8–35.9 kg). The sex distribution was balanced, with 28.5% intact males, 24.0% castrated males, 24.0% intact females, and 23.5% spayed females. The breed repartition is detailed in the table provided in the supplementary material (Supplementary Material 1).

### OTIS3 score

**Figure 1:**
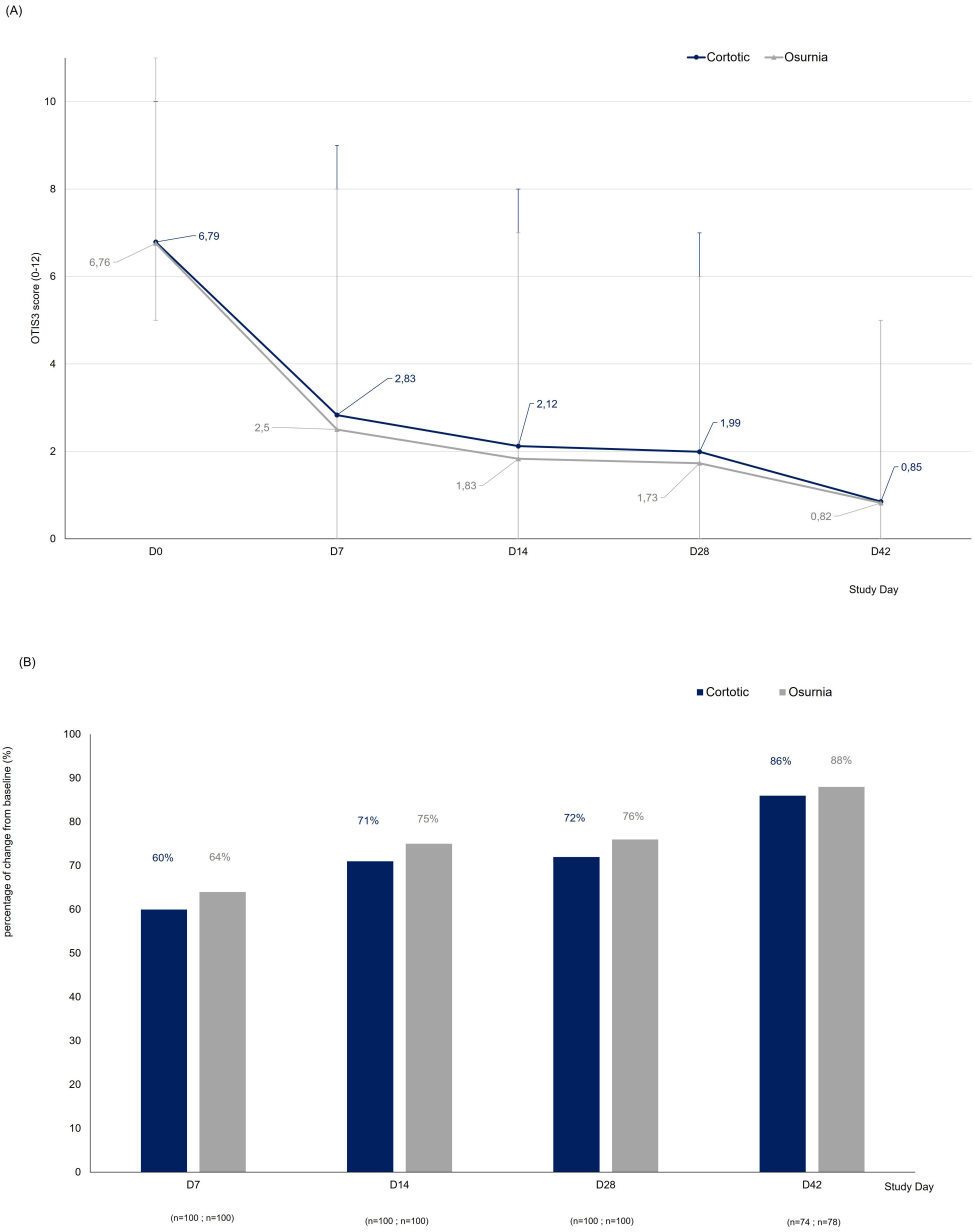
OTIS3 improvement over time: **(A)** mean OTIS3 scores evolution; **(B)** mean percentage of OTIS3 reduction from baseline.

A measurable decrease in OTIS3 scores was observed in both treatment groups (Figure 1).

The most substantial decrease was observed during the initial treatment phase at Day 7. In both groups, the difference in the mean OTIS3 score at D7 is significantly different to the mean OTIS3 score at D0 (*p* < 0.0001). However, the OTIS3 scores continued to improve throughout the study period.

In the Cortotic group, the mean OTIS3 score went from 6.79 (5–10) at D0 to 2.83 (0–9) at D7 (0–8), 2.12 (0–7) at D14 and 1.99 (0–5) at D28, remaining low at 0.85 at D42.

In the Osurnia group, the mean OTIS3 score was 6.76 (5–11) at D0, 2.50 (0–8) at D7, 1.83 (0–7) at D14, 1.73 (0–6) at D28 and 0.82 (0–5) at D42.

With an OTIS3 score improvement at D7 of 60% for Cortotic and 64% for Osurnia at D7, the OTIS improvement increased over time in both groups. The mean percent decrease from baseline in OTIS3 score showed no statistically significant difference between Cortotic and Osurnia groups at any of the study days (Figure 1).

At Day 28, the Cortotic group exhibited a mean percent decrease from baseline of 72% compared to a 76% reduction in the Osurnia group (*p* = 0.2396). The 95% confidence intervals (CI) for the mean percentage change were 67.0%−77.2% for Cortotic and 71.0%−81.2% for Osurnia. The mean treatment difference was −4.0%, with a 95% *CI* of −10.7% to 2.7%. Because the lower limit of the 95% *CI* (−10.7%) was greater than the pre-specified non-inferiority margin of −15%, the non-inferiority of Cortotic to Osurnia was established.

OTIS3 scores assessed 4 criteria (each criterium scored from 0 to 3) as clinical signs of otitis, i.e., erythema, oedema, discharge and erosion. Detailed analysis of each OTIS3 criterion revealed no significant differences in the reduction from baseline between the treatment groups at any study day (*p* ≥ 0.05). The similar mean baseline scores for each criterium in both groups confirmed the homogeneity in otitis severity at D0. The most prominent clinical signs were erythema (mean scores: Cortotic 2.11, Osurnia 2.12), discharge (mean scores: Cortotic 2.08, Osurnia 2.11) and oedema (mean scores: Cortotic 1.83, Osurnia, 1.75). Erosion scores were the lowest-rated criterion (Cortotic 0.77, Osurnia 0.78).

### Cytology evaluation

On Day 0, the baseline mean total cytologic score was 3.7 in the Cortotic group and 4.0 in the Osurnia group. The respective mean baseline scores for the specific cytologic categories were 1.1 and 1.3 for bacteria, 2.2 and 2.2 for yeast and 0.4 and 0.4 for inflammatory cells. Baseline semi-quantitative cytology of ear samples revealed a high prevalence of cases with bacteria as the sole microbiological finding (Cortotic: 64%, Osurnia: 66%), while *Malassezia* alone (Cortotic: 23%, Osurnia: 19%) and the co-occurrence of bacteria and *Malassezia* (Cortotic: 13%, Osurnia: 15%) were notably less frequent. Further microbiological analysis revealed a predominance of *Staphylococcus* spp. in both treatment groups (Cortotic: 69%; Osurnia: 68%). *Malassezia* spp. was less frequently identified (Cortotic: 35%; Osurnia: 34%). *Streptococcus* spp. accounted for 3% of isolates in the Cortotic group and 5% in the Osurnia group. Gram-negative bacteria, including *Proteus* spp. and *Pseudomonas* spp., were less prevalent (Cortotic: 3% for both, Osurnia: 3% and 5% respectively).

The analysis on the per protocol population demonstrated that the mean reduction from baseline for each cytologic score (bacteria, yeast, inflammatory cells), showed no statistically significant difference between the treatment groups at any of the study days.

**Figure 2:**
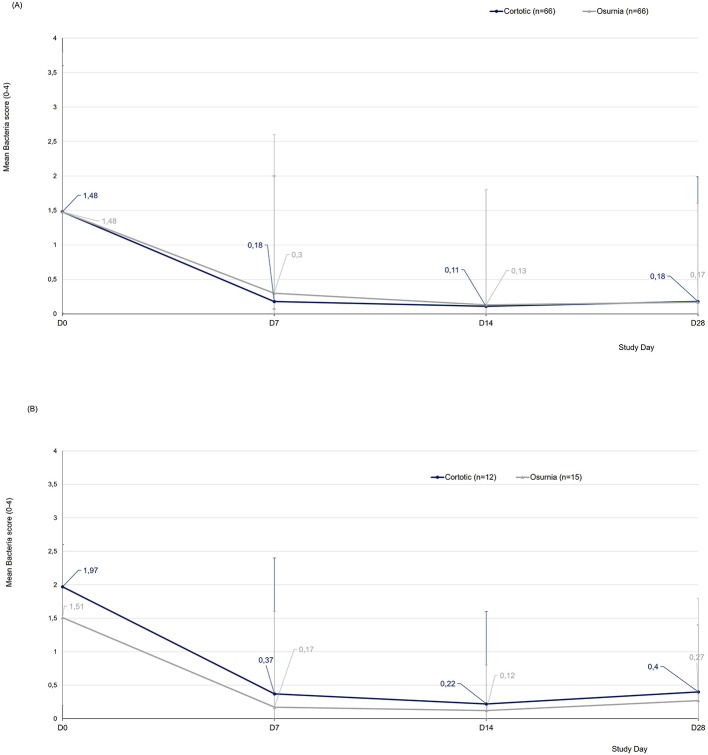
Mean bacteria score evolution over time for dogs with presence of bacteria at day 0: **(A)** dogs with exclusive presence of bacteria at day 0; **(B)** dogs with bacteria and yeast presence at day 0.

**Figure 3:**
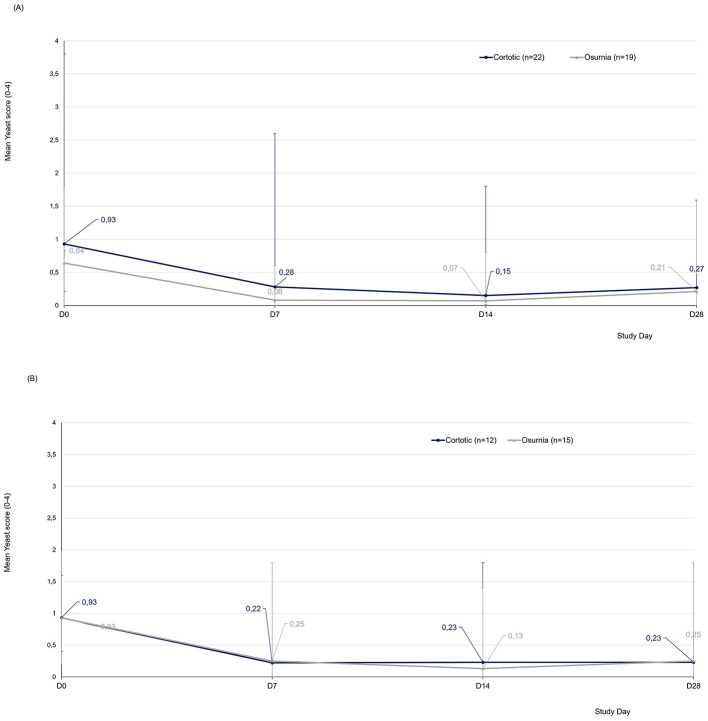
Mean yeast score evolution over time for dogs with presence of yeast at day 0: **(A)** dogs with exclusive presence of yeast at day 0; **(B)** dogs with bacteria and yeast presence at day 0.

To ensure clinical relevance, the longitudinal analysis of bacteria and yeast score reductions was restricted to the sub-populations of dogs testing positive for the respective pathogen at Day 0. The bacteria score reduction over time was evaluated in dogs presenting at Day 0 with either an exclusive bacterial infection or a mixed bacteria-yeast co-infection (Figure 2). In both study groups, the bacteria score decreased significantly by Day 7 compared to Day 0 (*p* < 0.0001). The bacteria score then stabilized at a minimal level (*p* < 0.2) through Day 28. Notably, this trend was consistent in subgroups of dogs presenting at Day 0 with either an exclusive bacterial infection or a mixed bacteria-yeast co-infection. The yeast score reduction over time was evaluated in dogs presenting at Day 0 with either an exclusive yeast infection or a mixed bacteria-yeast co-infection (Figure 3). At baseline, these dogs exhibited a minimal yeast load, which was reflected by uniformly low scores across both study groups. From Day 7, a statistically significant decrease from baseline in the mean yeast score was observed in both groups (*p* < 0.0001).

### Treatment success

**Figure 4:**
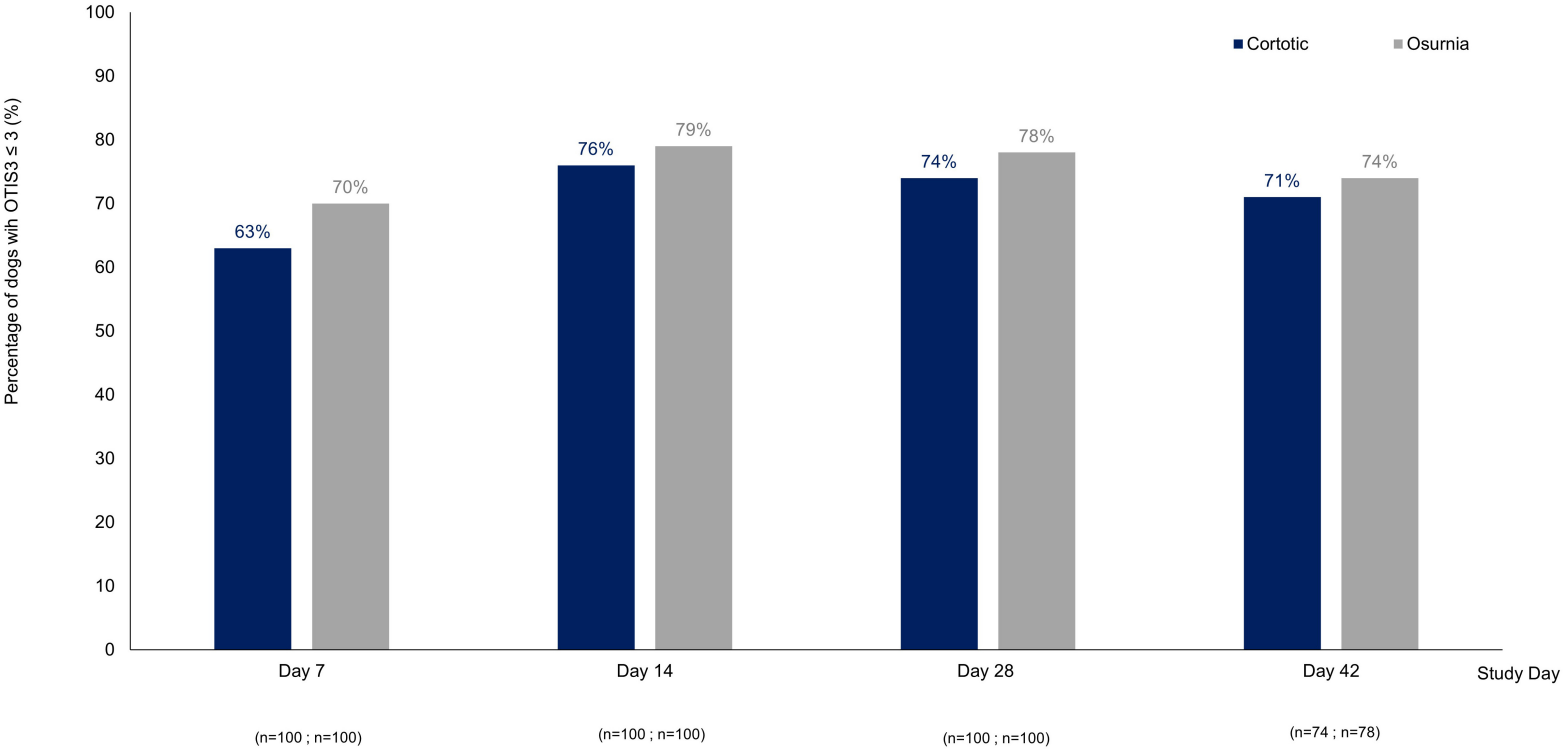
Success rate evolution over the time in each group.

Treatment success, defined as the percentage of dogs achieving an OTIS3 score ≤ 3, was evaluated in both groups at each study interval (Figure 4).

In the Cortotic group, the success rates were 63% at D7, 76% at D14, 74% at D28, and 71% at D42. In the Osurnia group, the success rates were 70% at D7, 77% at D14, 78% at D28, and 74% at D42. No statistically significant difference in success rates was observed between the treatment groups at any time point (respectively, *p* = 0.260, *p* = 0.635, *p* = 0.518, *p* = 0.618).

In the Cortotic group, 63% of dogs achieved clinical success by D7. Consequently, in accordance with the study protocol, only the remaining 37% of this group received an extended 14-day treatment regimen. In contrast, 100% of dogs in the Osurnia group completed the standard 14-day treatment regimen. Among the 37 dogs in the Cortotic group receiving the additional 7-day course, 14 achieved clinical success by D14, and 13 maintained or reached success by D28. Variations in the OTIS3 scores (shifting between 3 and 4) in several dogs accounted for the observed changes in clinical success status over these time points. Only one dog of the 63 dogs that achieved treatment success by Day 7 (OTIS3 score ≤ 3) without requiring an additional 7-day course of Cortotic™ exhibited an OTIS3 score of 4 at Day 14 and Day 28. An additional dog showed a OTIS3 of 3 at Day 14 and 4 at Day 28.

**Figure 5:**
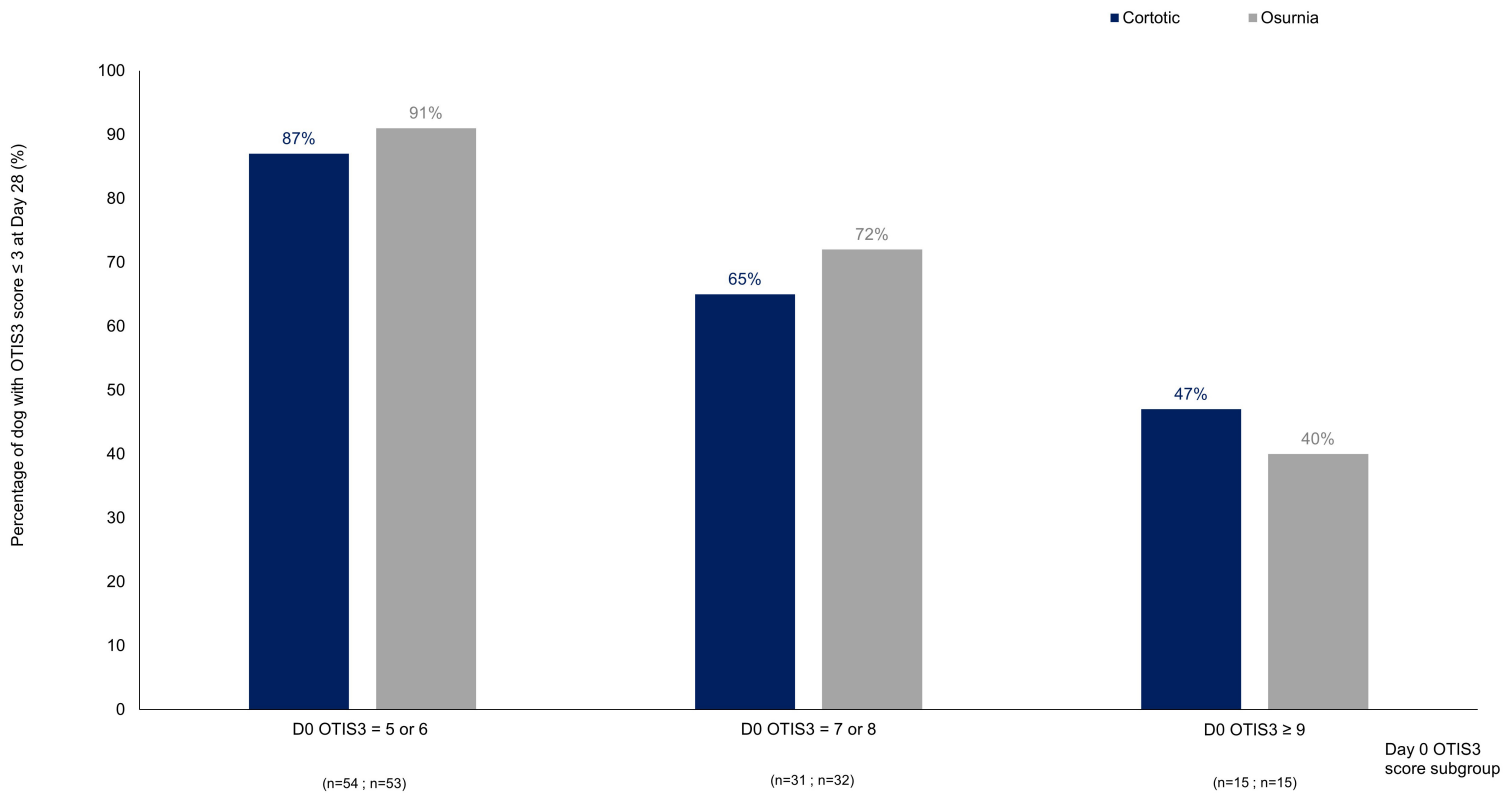
Success rate at day 28 depending on the OTIS3 score at day 0.

To evaluate the impact of baseline otitis severity on treatment outcome, the treatment success results were stratified into sub-groups based on the median baseline OTIS3 score of 6. A general trend was observed where the success rates declined in both treatment groups as the initial severity increased (Figure 5). In dogs with moderate initial severity (OTIS3 score of 5 or 6), Cortotic™ and Osurnia™ achieved a success rate of 87% and 91%, respectively. Both treatments maintained comparable success rates in cases of higher baseline severity (OTIS3 score of 7 or 8), at 65% for Cortotic™ and 72% for Osurnia™. Notably, in the most severe cases (OTIS3 score >9), Cortotic™ exhibited a success rate of 47% compared to 40% for Osurnia™. While less frequent, these cases are often more challenging to manage clinically.

### Relapse rate

The relapse rate was evaluated by calculating the percentage of dogs with an OTIS3 score ≥4 at Day 42 among those having a treatment success at Day 28. The relapse rate was 4% in the Cortotic group and 5% in the Osurnia group (*p* = 0.8254).

### Overall assessment of response to treatment

**Figure 6:**
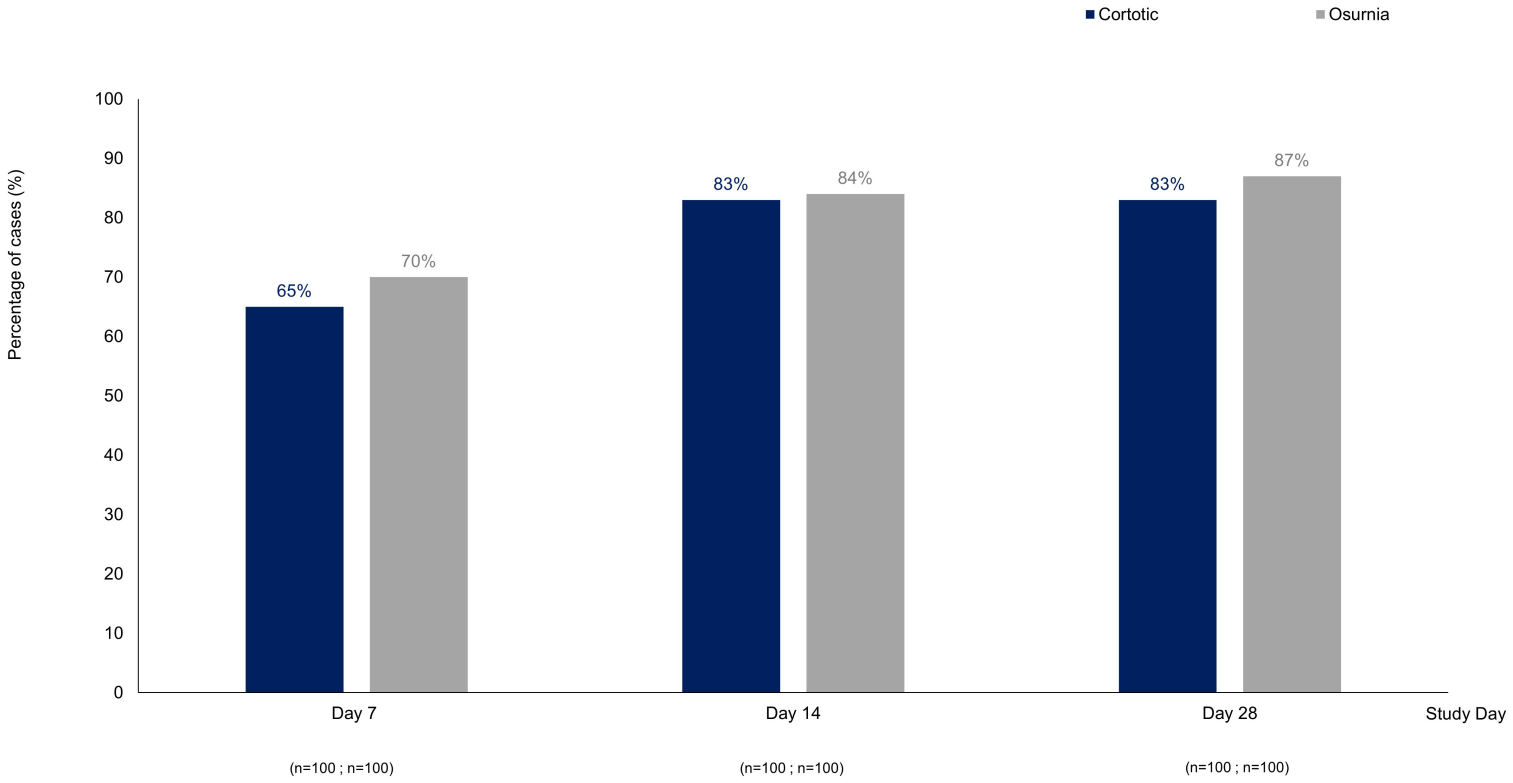
Distribution of treatment efficacy assessed as excellent or good by veterinarians.

Across all study time points, no statistically significant difference was found in overall assessment of treatment response between the two groups (Figure 6). On Day 28, the veterinarians subjectively assessed the efficacy as good or excellent in 83% of cases for Cortotic™ and 87% for Osurnia™. In both groups, considering the cases showing treatment success at Day 28, the veterinarians assessed the treatment response as good or excellent in 100% of cases.

### Safety evaluation

No local reactions were observed in either group. Two dogs in the Cortotic group and one dog in the Osurnia group experienced non-serious transient digestive disorders (vomiting, loose stools or diarrhea). These gastrointestinal signs were attributed to accidental ingestion of unusual food items. Both treatments were well-tolerated and demonstrated a positive safety profile.

## Discussion

This study compared two otic formulations, distinguished primarily by the absence of anti-infectious agents in Cortotic™. The results of this study demonstrate the non-inferiority of Cortotic™ compared to Osurnia™ with respect to the OTIS3 score improvement, cytological reduction and treatment success rate. By day 28, Cortotic™ administration resulted in a 72% reduction in the OTIS3 score and a treatment success of 74%, thereby confirming its efficacy as a first-line treatment for ECOE.

Complementing these objective parameters, the subjective assessments of treatment efficacy by attending veterinarians indicated comparable outcomes for both products, with high or marked efficacy reported at day 28 in 83% of Cortotic-treated cases and 87% of Osurnia-treated cases and 96% of dogs in both groups at day 42.

Both treatments demonstrated sustained efficacy, with clinical parameter improvements, particularly in OTIS3 scores, lasting through day 28 and up to day 42, notwithstanding the single-administration 7- or 14-days treatment. Relapse rates at day 42 were low for both treatments (4% for Cortotic™ and 5% for Osurnia™).

The study established a clear inverse correlation between the baseline OTIS3 score and the treatment success rate at Day 28 for both products, with success rates consistently decreasing as the initial severity of otitis increased. However, Cortotic™ demonstrated comparable success rates in dogs with moderate otitis and more remarkably also in the most severe cases. This sustained performance across the higher severity spectrum suggests that Cortotic™ maintains efficacy even in highly advanced disease. Nevertheless, the overall reduction in treatment success observed in the less common severe cases underscores the importance of rigorous follow-up by the attending veterinarian and the potential necessity for prolonged treatment to achieve complete resolution in dogs presenting with high initial OTIS3 scores.

In this study design, a key difference consisted in the treatment duration. The majority of Cortotic-treated dogs (63%) were considered clinically cured by day 7 and did not require further medication. In contrast, all Osurnia-treated dogs completed a full 14-day course.

Nonetheless, the sustained efficacy of Cortotic™ remained similar to the Osurnia™ efficacy with respectively 74% and 78% of treatment success at day 28. The difference in treatment duration could influence clinical outcomes. Only two dogs of the 63 dogs that received a 7-day course of Cortotic™ exhibited an OTIS3 score higher than 3 at Day 28, with both presenting a score of 4. A potential enhanced outcome cannot be excluded for dogs experiencing treatment failures at Day 28 in the Cortotic™ treatment group, had they benefited from an extended treatment regimen, potentially up to the full 14 days.

The veterinarians have a crucial role in assessing individual cases to explore extended ear treatment for enhanced therapeutic benefits. Additionally, while the OTIS3 score is a valuable objective tool, the predefined threshold of 3 for considering a dog cured in this study design might be too high for some individual cases. Further investigations into individualized Cortotic™ treatment durations would be helpful to further increase efficacy across all patient subsets.

This study revealed notable epidemiological observations within the examined population of client-owned dogs with otitis externa. The exclusive presence of bacteria constituted the majority of cases (65%), with a high prevalence of *Staphylococcus* spp., *Malassezia* spp. was isolated less frequently, representing the sole identified microorganism in 21% of cases. In this study, Cortotic™ demonstrated comparable efficacy to Osurnia™, regardless of the microbial population observed, despite the absence of antimicrobial agents in its formulation. These findings are consistent with a recent European study, which reported a high number of cases with *Malassezia* only (7). The cytological analysis provides evidence that the topical administration of a solely corticosteroid (hydrocortisone aceponate) may ameliorate the ear dysbiosis without the need for antimicrobials in most of the cases in first line treatment. Further investigations employing high-throughput sequencing would be valuable to elucidate the direct impact of topical treatment on the ear microbiota and mycobiota (13). A potential limitation of this study is its single-country design, as the presented epidemiological data are representative of the Japanese canine population. However, this limitation may be mitigated by the potential to extrapolate efficacy findings for specific cytological categories, even if their prevalence exhibits geographical variations (3, 5, 7). The objective was to confirm first observations in Europe, compared to a different product, and in another region of the world, to alleviate the effects of potential geographical variations in the clinical and microbial aspects of otitis externa in dogs.

Differing product administration protocols, as per recommended in the labels, could constitute another potential limitation. The daily application of Cortotic™ vs. the single-dose administration of Osurnia™, coupled with the variation in treatment duration, may have introduced bias. However, this limitation is somewhat mitigated by the similar results reported in the study by Rigaut et al. (7), which compared Cortotic™ and Surolan™, both daily administration products (7). The recent introduction of this novel canine otitis treatment approach, using exclusively a corticosteroid (hydrocortisone aceponate), into veterinary medicine necessitates further trials in broader clinical settings and ongoing observation of its efficacy in practical applications. To confirm its positioning as a possible first line treatment for canine otitis externa, findings comparing it to another combination product containing different antimicrobials and with a different protocol of administration was one of the objectives of this current study.

Effective long-term management of recurrent ECOE necessitates addressing both the underlying etiological factors and the consequential inflammation. The repeated exposure to low concentrations of antibiotics has been recognized as one of the factors to increase the risk for development of multi-resistant bacterial isolates (14–16). The rise of antimicrobial resistance and the potential impact on ear microbiome have driven the evaluation of non-antibiotic alternatives for canine otitis externa (13, 17, 18). Because canine otitis externa is an inflammatory condition rather than a primary infection, an anti-inflammatory-only treatment strategy could control inflammation and consequently help to resolve secondary overgrowth.

The present study lends further support to the significance of the initial inflammatory cascade in the pathogenesis of ECOE (2) and underscores the importance of targeting inflammation for effective management and rapid resolution of clinical signs (13, 19). In numerous cases of non-purulent otitis externa, the attenuation of inflammation alone can be sufficient to promote substantial clinical and microbiological improvement. Hydrocortisone aceponate (HCA), the active pharmaceutical ingredient in Cortotic™, is a di-ester corticosteroid characterized by its stability, potency, and lipophilicity. These physicochemical properties contribute to HCA's enhanced efficacy within the dermis, the primary site of inflammation in otitis externa, while optimizing its safety through metabolization in the skin leading to systemic elimination of very low activity metabolites (20). The advantageous pharmaceutical profile of HCA has been substantiated through extensive clinical application, particularly in the treatment of otitis externa and canine atopic dermatitis (5, 7, 21–23). The findings of this present study support the evaluation of a topical treatment containing only corticosteroids as a primary approach before considering antimicrobial therapy. In light of increasing antimicrobial resistance, the exclusive use of this di-ester anti-inflammatory agent offers an innovative therapeutic approach (1, 24, 25). To determine the most appropriate treatment strategy for ECOE, both a comprehensive baseline clinical assessment and diligent veterinary follow-up remain essential. The ECOE treatment primarily aims for the rapid control of inflammation and the resolution of secondary microbial overgrowth. In the absence of parasitic infestation, the first-line treatment of ECOE, especially when accompanied by clinical signs of canine atopic dermatitis, could prioritize the exclusive use of topical corticosteroids (1).

## Conclusions

For treating ECOE in dogs, Cortotic™ (solely hydrocortisone aceponate di-ester) demonstrated clinical and cytological improvements, along with veterinarian efficacy assessments, comparable to those of Osurnia™ (betamethasone acetate, florfenicol, and terbinafine). These findings support Cortotic™ integration into the first-line therapeutic options in external otitis management. The introduction of Cortotic™ into veterinary medicine opens up positive and promising therapeutic options.

## Data Availability

The raw data supporting the conclusions of this article will be made available by the authors, without undue reservation.
