# Supplementary material for: Treatment of canine otitis externa: a randomized, double-blinded, clinical field study comparing the hydrocortisone aceponate monotherapy to a multi-agent reference product
Source: Front Vet Sci. 2026 Jul 28;13:1861746. doi: 10.3389/fvets.2026.1861746 (PMC13457320; doi:10.3389/fvets.2026.1861746)
Supplement: Supplementary file 1 [file Data_Sheet_1.pdf]

Supplementary Material 1 : distribution of enrolled dogs

		Both group (N)	Both group (%)	Cortotic group (N)	Cortotic group (%)	Osurnia group (N)	Osurnia group (%)
Breed							
Dog breed (Purebreed)	Poodle (Toy)	34	17.0	20	20.0	14	14.0
	Dachshund (Miniature)	20	10.0	9	9.0	11	11.0
	Shiba Inu	9	4.5	7	7.0	2	2.0
	Cavalier King Charles Spaniel	5	2.5	3	3.0	2	2.0
	Golden Retriever	5	2.5	2	2.0	3	3.0
	Labrador Retriever	5	2.5	3	3.0	2	2.0
	American Cocker Spaniel	3	1.5	1	1.0	2	2.0
	Pembroke Welsh Corgi	3	1.5	1	1.0	2	2.0
	Pug	3	1.5	1	1.0	2	2.0
	Jack Russell Terrier	2	1.0	0	0.0	2	2.0
	Chihuahua	2	1.0	1	1.0	1	1.0
	Beagle	2	1.0	0	0.0	2	2.0
	French Bulldog	2	1.0	0	0.0	2	2.0
	Border Collie	2	1.0	1	1.0	1	1.0
	Miniature schnauzer	2	1.0	1	1.0	1	1.0
	Kishu	1	0.5	0	0.0	1	1.0
	Shih Tzu	1	0.5	1	1.0	0	0.0
	Shetland Sheepdog	1	0.5	0	0.0	1	1.0
	Bernese Mountain Dog	1	0.5	1	1.0	0	0.0
	Papillon	1	0.5	0	0.0	1	1.0
	Boston Terrier	1	0.5	0	0.0	1	1.0
	Maltese	1	0.5	1	1.0	0	0.0
	Yorkshire Terrier	1	0.5	1	1.0	0	0.0
	SUBTOTAL Purebreed	107	53.5	54	54.0	53	53.0
Mixed-breed	SUBTOTAL Mixed-breed	93	46.5	46	46.0	47	47.0
Sex							
	Male	57	28.5	29	29	28	28
	Neutered male	48	24.0	25	25	23	23
	Female	48	24.0	24	24	24	24
	Neutered female	47	23.5	22	22	25	25
Age (years)							
	Mean	4.6		4.6		4.5	
	Min	1.0		1.0		1.0	
	Max	12.0		10.0		12.0	
Body Weight (kg)							
	Mean	10		10		10	
	Min	1.8		1.8		1.8	
	Max	35.9		35.9		35.2	
